# Morphophysiological Responses of Black Pepper to GA_3_: Growth, Photosynthesis, Carbohydrates and Flowering

**DOI:** 10.3390/ijms27093932

**Published:** 2026-04-28

**Authors:** Marcos Antônio Cezario Dias, Vinicius de Souza Oliveira, Fernando Gomes Hoste, Ana Júlia Câmara Jeveaux Machado, Janyne Soares Braga Pires, Francine Bonomo Crispim Silva, Bliane Morozini Bacheti, Geraldo Rogério Faustini Cuzzuol, Carla da Silva Dias, Lúcio de Oliveira Arantes, Edilson Romais Schmildt, Sara Dousseau-Arantes

**Affiliations:** 1 Departamento de Ciências Biológicas, Centro de Ciências Humanas e Naturais, Universidade Federal do Espírito Santo, Avenida Fernando Ferrari, 514, Goiabeiras, Vitória CEP 29075-910, ES, Brazil; marcosantonio10045@gmail.com (M.A.C.D.); fernandohost@gmail.com (F.G.H.); anajucamara@gmail.com (A.J.C.J.M.); geraldo.cuzzuol@ufes.br (G.R.F.C.); 2 Centro de Pesquisa, Desenvolvimento e Inovação Norte, Instituto Capixaba de Pesquisa, Assistência Técnica e Extensão Rural, Caixa Postal 62, Linhares CEP 29900-970, ES, Brazil; bliane.bacheti@gmail.com (B.M.B.); carla.dias@incaper.es.gov.br (C.d.S.D.); lucio.arantes@incaper.es.gov.br (L.d.O.A.); 3 Departamento de Ciências Agrárias e Biológicas, Centro Universitário Norte do Espírito Santo, Universidade Federal do Espírito Santo, BR101 Norte, Km. 60, Bairro Litorâneo, São Mateus CEP 29932-540, ES, Brazil; janynesbraga@hotmail.com (J.S.B.P.); francine.b.silva@edu.ufes.br (F.B.C.S.); e.romais.s@gmail.com (E.R.S.)

**Keywords:** biomass, carbohydrates, flowering, growth, phytohormones, *Piper nigrum*

## Abstract

Black pepper (*Piper nigrum* L.) faces challenges related to irregular flowering, which compromises crop productivity. Gibberellic acid (GA_3_) is a plant growth regulator known for its role in inducing reproductive processes, although its effects on this species are not yet fully understood. This study aimed to evaluate the influence of different GA_3_ doses on flowering and vegetative growth in black pepper plants. The experiment was conducted with black pepper seedlings of the Bragantina cultivar in a randomized block design, with four doses of GA_3_ (0, 10, 20, and 30 mg L^−1^) and six replications, using eight-month-old plants grown in pots under full sun. GA_3_ applications were performed in two floral induction cycles. Variables related to flowering, chlorophyll a fluorescence, vegetative growth, biomass allocation, and carbohydrate distribution were evaluated. The data were subjected to analysis of variance, regression analysis, mean grouping tests, and principal component analysis. The results showed that intermediate doses (10 and 20 mg L^−1^) significantly stimulated flowering at early developmental stages, whereas the 30 mg L^−1^ dose enhanced vegetative growth while reducing floral induction. Additionally, GA_3_ affected physiological parameters by increasing photosynthetic efficiency and altering carbohydrate balance, with higher accumulation of soluble sugars in leaves and reduced starch content in roots. It is concluded that GA_3_ application is a promising strategy to modulate reproductive transition in black pepper, with 10 to 20 mg L^−1^ doses recommended to promote flowering without compromising plant development.

## 1. Introduction

Black pepper (*Piper nigrum* L.) is one of the most traded spices in the world, with Brazil occupying the second position in global production [1]. Espírito Santo stands out as the largest national producer, representing about 60% of the country’s production [2]. Despite its economic relevance, the crop faces challenges, such as uneven fruiting, with flowering and fruiting being phenological stages influenced by various environmental and physiological factors and decisive for productivity [3,4,5].

Factors such as plant age, environmental conditions, genetic characteristics, and morphological characteristics and photosynthesis are the main key factors for crop flowering [6,7]. In the study region, an increase in fruit set failures is also observed, apparently due to extreme high temperatures. This occurs because, at high temperatures, there is an alteration in the sexual expression of the flowers, and the male part does not form correctly, reducing the number of hermaphrodite flowers, which leads to reduced pollination and fruit set failure in black pepper [8].

Among the alternatives for standardizing flowering, the use of gibberellic acid (GA_3_), a phytohormone that regulates several processes related to plant growth and development, stands out. Natural gibberellins are a class of plant hormones whose synthesis is related to the terpenoid wheel and which have functions in plant metabolism, being responsible for stem elongation, pollen production, pollen tube development, fruit and seed growth, seed germination, sex determination, and the transition from the vegetative to the reproductive phase [9]. In some species, GA_3_ has been widely used to influence plant development and reproduction [10,11,12], advance flowering and increase production [13,14,15,16], and stimulate vegetative growth [17].

Although studies characterizing the factors related to black pepper flowering are scarce, experiments conducted in India identified gibberellin as the main hormone related to the flowering process [3]. Also, the use of GA_3_ in black pepper plants at the juvenile stage, where flowering began three years after the exogenous application of the growth regulator, was tested by Gusta et al. [18] through foliar spraying at a concentration of 10 mg L^−1^. It is noteworthy that the use of GA_3_ positively influenced flowering with a significant increase in the number of flowers; however, early flowering was not observed in the plants, a fact that may be related to the low concentration of GA_3_ to which the plants were subjected. Conversely, very high doses of GA_3_ can compromise root development, reducing the specific root length and the mass fraction allocated to the root system [19,20,21,22]. Thus, further studies are needed to investigate the influence of gibberellins on black pepper flowering.

Therefore, this study sought to evaluate the effects of GA_3_ on the standardization of black pepper flowering, analyzing its impact on vegetative development, photochemical efficiency, biomass allocation, and carbohydrate metabolism.

## 2. Results

### 2.1. Flowering and Phenological Classification

**Figure 1:**
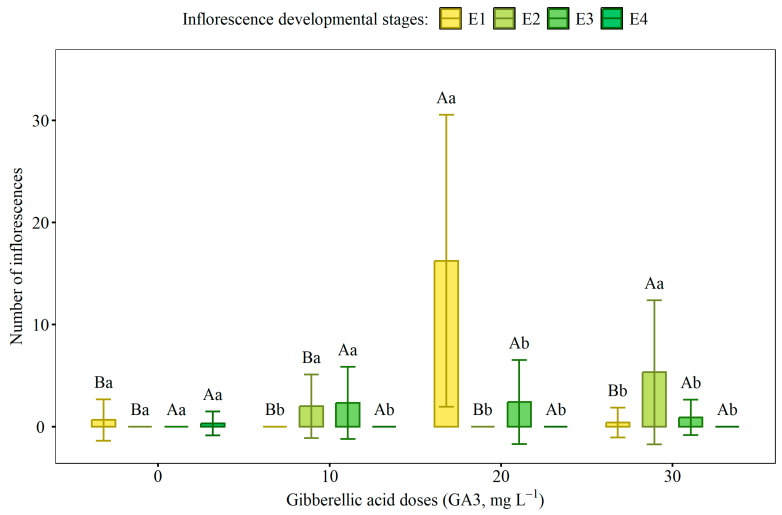
Influence of different doses of gibberellic acid (GA_3_) on the number of black pepper inflorescences at four flower development stages: E1 (immature spikes), E2 (spikes with flowers), E3 (spikes with immature berries), and E4 (fully mature spikes with green berries). Bars indicate the standard errors of the means of 6 replicates of 14 plants. Capital letters compare GA doses within each development stage, while lowercase letters compare development stages within each dose. Equal letters indicate no significant differences by the Scott–Knott test (*p* ≤ 0.05).

The number of inflorescences was significantly influenced by the interaction between GA_3_ doses and flower development stages (Figure 1 and Table A1). The highlight was the 20 mg L^−1^ dose, which promoted a significant increase in the number of inflorescences at the E1 stage (immature spikes), reaching an average of over 15 inflorescences per plant, a significantly higher value compared to the other stages at the same dose and the other treatments. In contrast, this same dose resulted in one of the lowest numbers of inflorescences at the E3 stage (spikes with immature berries). The application of 10 mg L^−1^ reduced flower production at the E2 (spikes with flowers) and E3 stages, an effect similar to that observed at the 30 mg L^−1^ dose for the E2 stage. In the control (0 mg L^−1^), all stages showed low flower formation, with no significant differences between them.

### 2.2. Chlorophyll a Fluorescence

**Figure 2:**
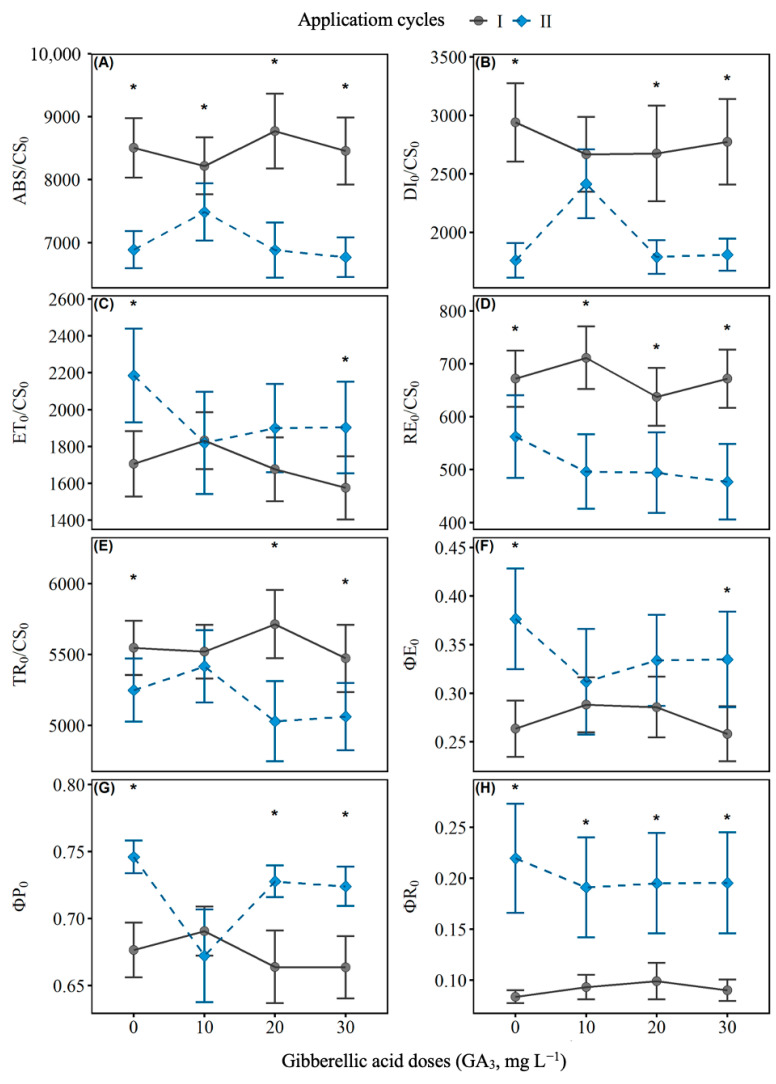
Effects of different doses of GA_3_ on chlorophyll a fluorescence parameters in black pepper leaves (*Piper nigrum* L.), cultivar Bragantina, evaluated in two application cycles. The variables analyzed were: (**A**) ABS/CS_0_ (energy absorption by reaction center), (**B**) DI_0_/CS_0_ (energy dissipation), (**C**) ET_0_/CS_0_ (electron transport rate), (**D**) RE_0_/CS_0_ (final reduction in the electron transport chain), (**E**) TR_0_/CS_0_ (energy capture by the reaction center), (**F**) ΦE_0_ (electron transport efficiency to plastoquinone), (**G**) ΦP_0_ (quantum efficiency of photosystem II), and (**H**) ΦR_0_ (reduction efficiency of final acceptors in the electron transport chain). The curves represent the first (●) and second (◆) application cycles at doses of 0, 10, 20 and 30 mg L^−1^ of GA_3_. Error bars indicate the standard errors of the means. Asterisks indicate statistically significant differences between doses within each cycle, as per the Student’s *t*-test (*p* ≤ 0.05).

The application of GA_3_ resulted in significant changes in chlorophyll a fluorescence parameters in leaves of black pepper, cultivar Bragantina, with variations between application cycles (Figure 2 and Table A2). In the first cycle, there was an increase in the efficiency of light energy capture and transport observed by the increases in ABS/CS_0_ (A), DI_0_/CS_0_ (B), RE_0_/CS_0_ (D), and TR_0_/CS_0_ (E) values with increasing GA_3_ doses, while in the second cycle there was a reduction or stabilization of these parameters. Adjustments in the electron transport chain flux were observed in the second cycle with an increase in quantum yields (ΦP_0_ and ΦE_0_) compared to the first cycle.

**Figure 3:**
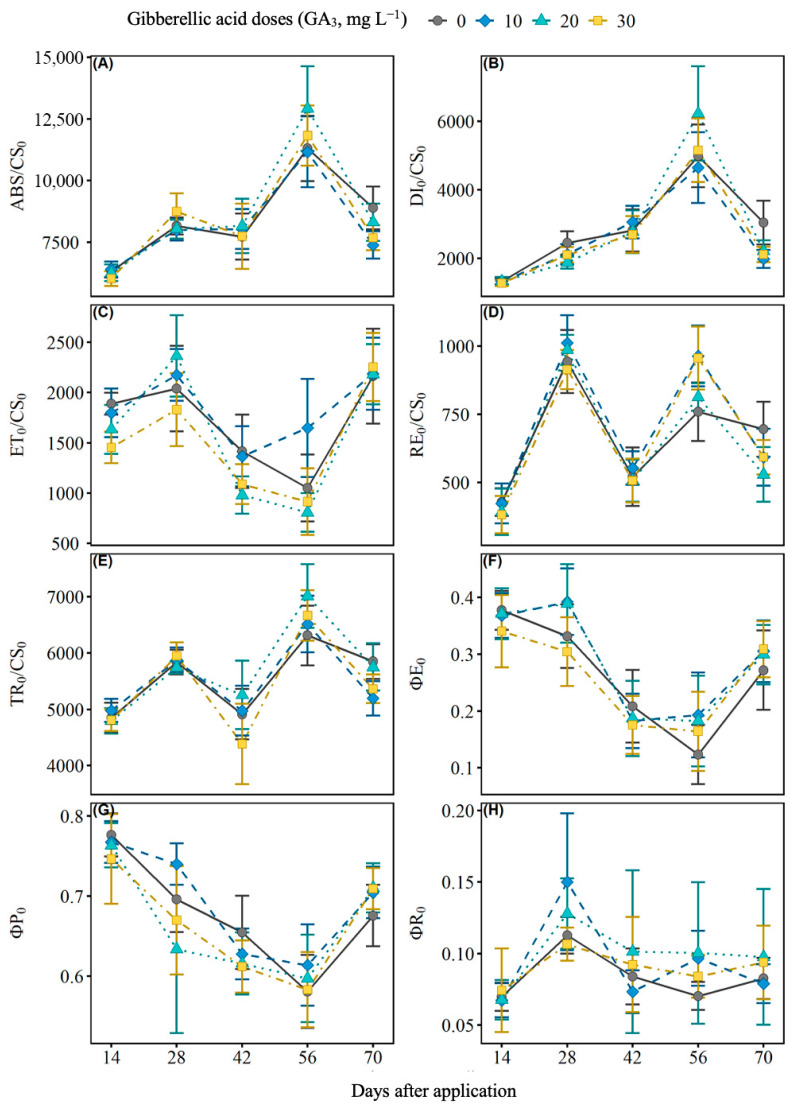
Effects of different doses of gibberellic acid (GA_3_) on chlorophyll a fluorescence parameters in black pepper leaves (*Piper nigrum* L.), cultivar Bragantina, evaluated over time. The variables analyzed were: (**A**) ABS/CS_0_ (energy absorption per reaction center), (**B**) DI_0_/CS_0_ (energy dissipation per reaction center), (**C**) ET_0_/CS_0_ (electron transport rate beyond QA^−^), (**D**) RE_0_/CS_0_ (reduction of final acceptors in the electron transport chain), (**E**) TR_0_/CS_0_ (energy capture by the reaction center), (**F**) ΦE_0_ (quantum efficiency of electron transport), (**G**) ΦP_0_ (quantum efficiency of primary photochemistry), and (**H**) ΦR_0_ (proportion of active reaction centers). The curves represent the doses of 0, 10, 20, and 30 mg L^−1^ of GA_3_, evaluated at 14, 28, 42, 56, and 70 days after application. Error bars indicate the standard errors of the means.

GA_3_ application significantly influenced chlorophyll a fluorescence over time (Figure 3 and Table A2). The absorption (ABS/CS_0_) and energy dissipation (DI_0_/CS_0_) fluxes increased up to 56 days after application, with a subsequent reduction at 70 days (Figure 3A,B). Energy capture (TR_0_/CS_0_) showed a similar pattern (Figure 3E), while electron transport (ET_0_/CS_0_) decreased up to 42 days, followed by recovery at 70 days (Figure 3C). The quantum yields, ΦP_0_ (primary photochemistry) and ΦE_0_ (electron transport), decreased up to 42 days after application, with a subsequent increase (Figure 3F,G). This behavior was more evident in plants treated with 10 and 20 mg L^−1^ of GA_3_, which showed greater efficiency in the use of light energy compared to the control and the 30 mg L^−1^ dose. Furthermore, differences between doses were evident at certain evaluation times. For the ΦR_0_ parameter, variations between treatments can be observed at 42 and 56 days after application (Figure 3H).

### 2.3. Chlorophyll Index

**Figure 4:**
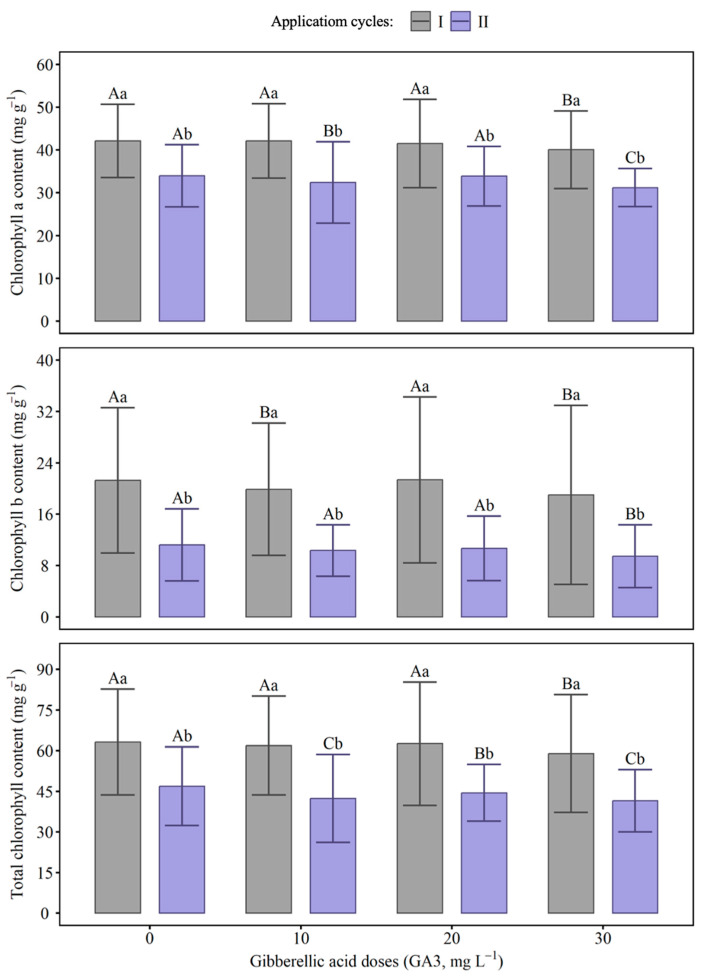
Chlorophyll a, chlorophyll b, and total chlorophyll concentrations in response to different doses of gibberellic acid (GA_3_) in cycles I and II. Bars represent standard errors. Capital letters compare doses between cycles, while lowercase letters compare doses within each cycle. Means followed by the same letter do not differ from each other by the Scott–Knott test (*p* ≤ 0.05).

The application of GA_3_ resulted in variations in chlorophyll indices over time and between the evaluated cycles (Figure 4 and Table A3). In cycle I, the values of chlorophyll a, chlorophyll b, and total chlorophyll were higher compared to cycle II, regardless of the applied doses. Chlorophyll concentrations fluctuated in response to GA_3_ doses, with a reduction observed at the 30 mg L^−1^ dose in cycle II. In cycle I, the application of GA_3_ did not affect chlorophyll levels.

**Figure 5:**
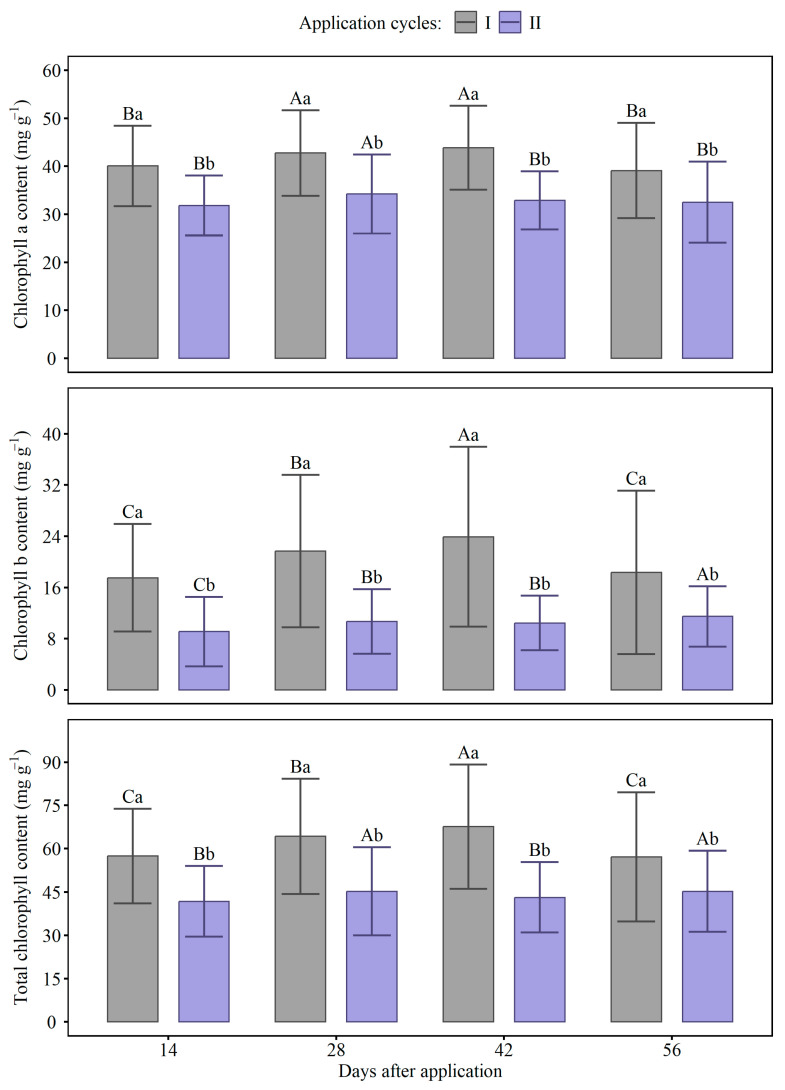
Chlorophyll a, chlorophyll b and total chlorophyll concentrations on different days after gibberellic acid (GA_3_) application in cycles I and II. Bars represent standard errors. Capital letters compare cycles within each day after application, while lowercase letters compare days within each cycle. Means followed by the same letter do not differ from each other by the Scott–Knott test (*p* ≤ 0.05).

Over the days following application, chlorophyll A and B and total chlorophyll levels fluctuated in both evaluation cycles, with no clear trend toward continuous decline (Figure 5 and Table A3). In cycle I, levels were consistently higher than in cycle II at virtually all time points analyzed, with statistically significant differences. Chlorophyll B was the pigment that showed the greatest variation, especially in cycle I, with an increase at 42 days. In cycle II, values remained lower and relatively stable over time. Total chlorophyll followed a similar pattern to chlorophyll A and B, reflecting the predominance of higher values in cycle I. The letters indicate significant interactions between cycles and time points, highlighting the superiority of cycle I, especially at 28 and 42 days after application.

### 2.4. Growth and Biomass Allocation Analyses

**Figure 6:**
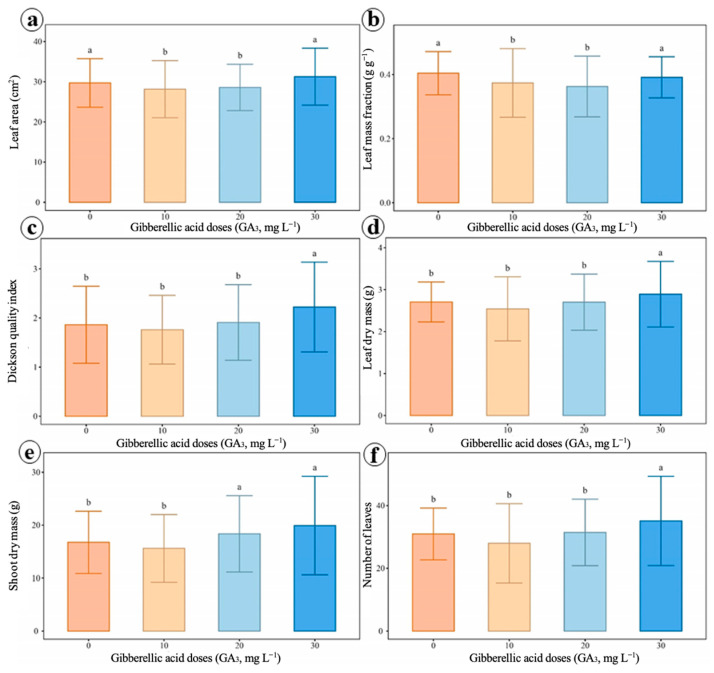
Effects of different doses of gibberellic acid (GA3) on leaf characteristics of black pepper, cv. Bragantina. (**a**) Leaf area. (**b**) Leaf mass fraction. (**c**) Dickson quality index. (**d**) Leaf dry mass. (**e**) Shoot dry mass. (**f**) Number of leaves. Means followed by distinct letters indicate significant differences between treatments by the Scott–Knott test (*p* < 0.05). Error bars represent the standard deviations.

Treatments with gibberellic acid positively influenced the morphological variables evaluated (Figure 6 and Table A4). An increase in total leaf area (Figure 6a), leaf mass fraction (Figure 6b), Dickson quality index (Figure 6c), leaf dry mass (Figure 6d), shoot dry mass (Figure 6e), and leaf number (Figure 6f) was observed in plants treated with 30 mg L^−1^ of GA_3_, which presented the highest values compared to the control.

**Figure 7:**
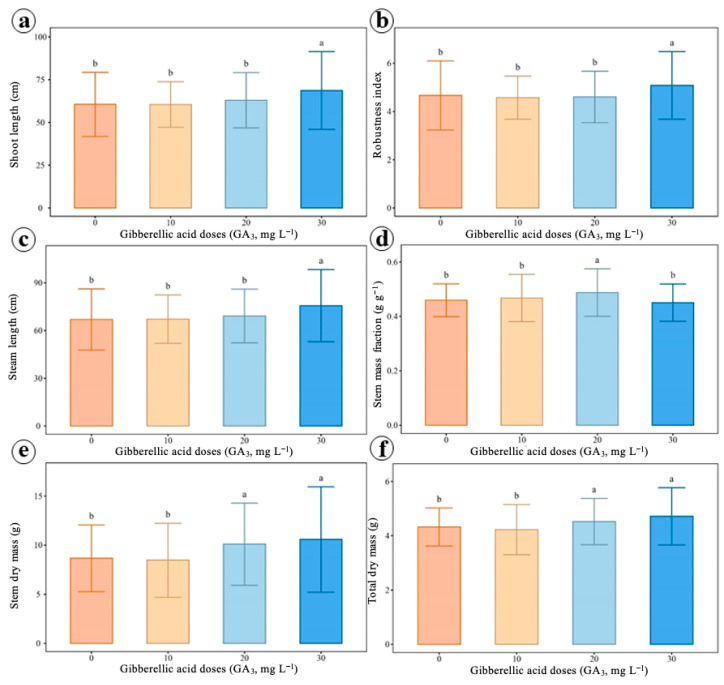
Effects of different doses of gibberellic acid (GA) on growth characteristics of black pepper, cv. Bragantina. (**a**) Shoot length. (**b**) Robustness index. (**c**) Stem length. (**d**) Stem mass fraction. (**e**) Stem dry mass. (**f**) Total dry mass. Means followed by distinct letters indicate significant differences between treatments by the Scott–Knott test (*p* < 0.05). Error bars represent the standard deviations.

The variables related to stem growth were significantly influenced by the gibberellic acid doses (Figure 7 and Table A4). An increase in shoot length (Figure 7a), robustness index (Figure 7b), stem length (Figure 7c), stem mass fraction (Figure 7d), stem dry mass (Figure 7e) and total dry mass (Figure 7f) was observed, the dose of 30 mg L^−1^ providing the highest values. The other variables, such as cutting length and specific stem length, did not present significant differences between the treatments.

**Figure 8:**
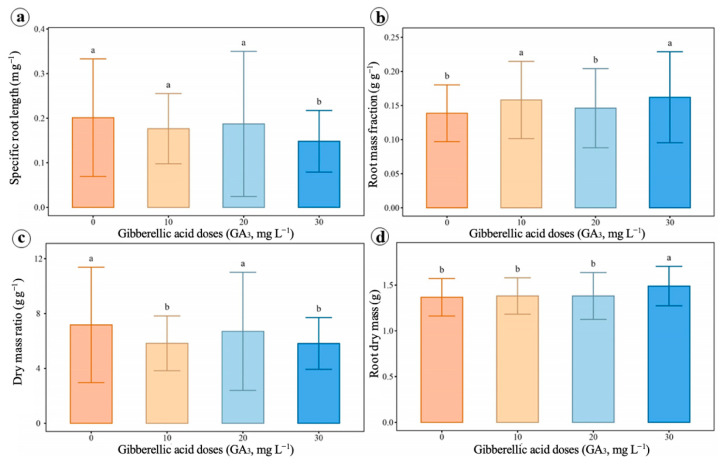
Effects of different doses of gibberellic acid (GA3) on root characteristics of black pepper, cv. Bragantina. (**a**) Specific root length. (**b**) Root mass fraction. (**c**) Dry mass ratio. (**d**) Root dry mass. Means followed by distinct letters indicate significant differences between treatments by the Scott–Knott test (*p* < 0.05). Error bars represent the standard deviations.

GA_3_ application stimulated black pepper root growth, especially at doses of 10 and 30 mg L^−1^ (Figure 8 and Table A4). At these concentrations, increases in root mass fraction (Figure 8b) and root dry mass (Figure 8d) were observed compared to the control. Furthermore, the 30 mg L^−1^ dose significantly reduced the shoot dry mass-to-root dry mass ratio (Figure 8c), indicating greater investment in root system growth. On the other hand, specific root length (Figure 8a) did not show significant differences among most treatments. The number of roots, root length, root fineness, root volume, and root tissue density did not show significant differences among treatments.

**Figure A1:**
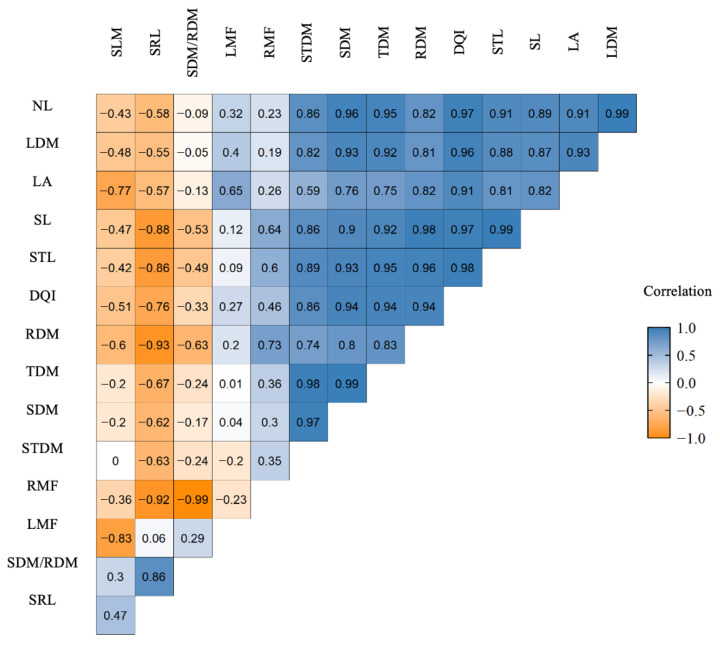
Correlation matrix for variables related to functional and structural characteristics of plants treated with GA_3_. The analyzed variables include: number of leaves (NL), leaf dry mass (LDM), leaf area (LA), stem length (SL), shoot length (STL), Dickson quality index (DQI), root dry mass (RDM), total dry mass (TDM), shoot dry mass (SDM), stem dry mass (STDM), root mass fraction (RMF), leaf mass fraction (LMF), SDM/RDM, stem mass fraction (SLM) and specific root length (SRL). Correlation values are represented by a color scale, where shades of blue indicate positive correlations and shades of orange indicate negative correlations.

The correlation matrix indicated significant correlation patterns between the variables evaluated in plants treated with GA_3_ (Figure A1). The correlation between leaf number and leaf dry mass (0.99), total leaf area (0.96), and the Dickson quality index (0.97) was strongly positive. Leaf dry mass also correlated positively with leaf area (0.93) and DQI (0.96). Stem length correlated strongly with shoot length (0.99) and DQI (0.98). Root mass fraction correlated negatively with the shoot dry mass-to-root dry mass ratio (SDM/RDM) (−0.99) and with specific root length (−0.92). Furthermore, leaf area correlated negatively with stem mass fraction (−0.77) and SRL (−0.57). Shoot length also correlated negatively with specific root length (−0.86).

**Figure 9:**
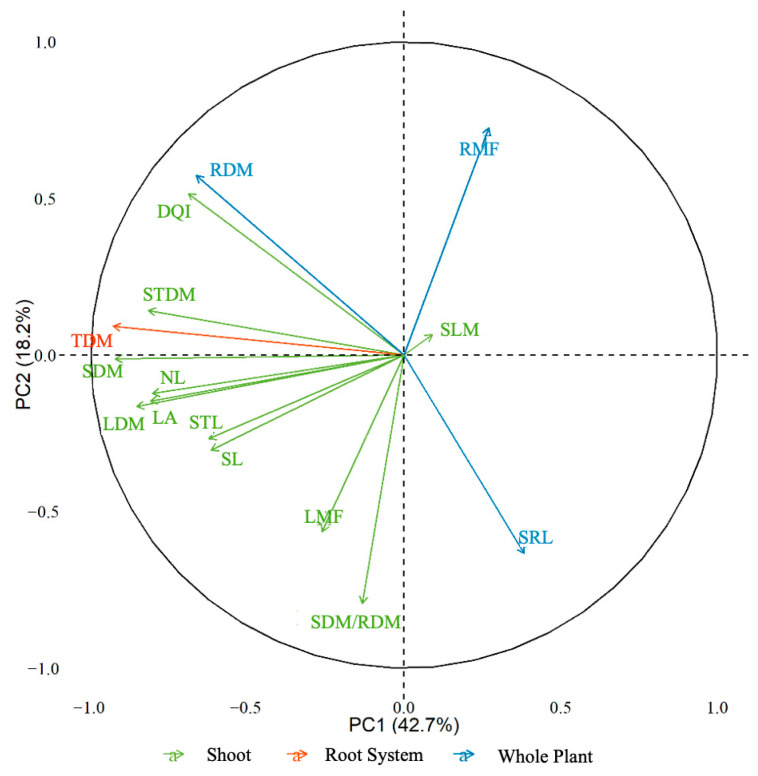
Principal component analysis (PCA) representing the relationships between variables associated with the shoot, root system, and the plant as a whole in plants treated with GA3. The PC1 (42.7%) and PC2 (18.2%) axes represent the explained variance. The arrows indicate the evaluated variables: number of leaves (NL), leaf dry mass (LDM), leaf area (LA), stem length (SL), shoot length (STL), Dickson quality index (DQI), root dry mass (RDM), total dry mass (TDM), shoot dry mass (SDM), stem dry mass (STDM), root mass fraction (RMF), leaf mass fraction (LMF), stem mass fraction (SLM), SDM/RDM ratio, and specific root length (SRL). The direction and length of the arrows reflect the contribution of the variables to the principal components.

Principal component analysis (PCA) (Figure 9) showed that the first two axes explained 60.9% of the total data variability, with 42.7% attributed to the first principal component (PC1) and 18.2% to the second principal component (PC2). The analyzed variables were categorized into three groups: shoot (green), root system (red), and whole plant (blue).

The variables associated with the shoot were most closely related and contributed most to the variation in PC1. Among these variables, the following stand out: number of leaves (NL), leaf dry mass (LDM), leaf area (LA), and stem length (SL). The variables related to the root system showed less influence on the separation of components, with shorter vectors distributed closer to the origin.

The variables related to the whole plant presented longer vectors, indicating a greater influence in explaining the data variability. The root efficiency coefficient (SRL) and root mass fraction (RMF) were the variables most associated with this group. The distribution of vectors shows the relationship between the variables and their contribution to the differentiation of the principal components.

### 2.5. Non-Structural Carbohydrates

**Figure 10:**
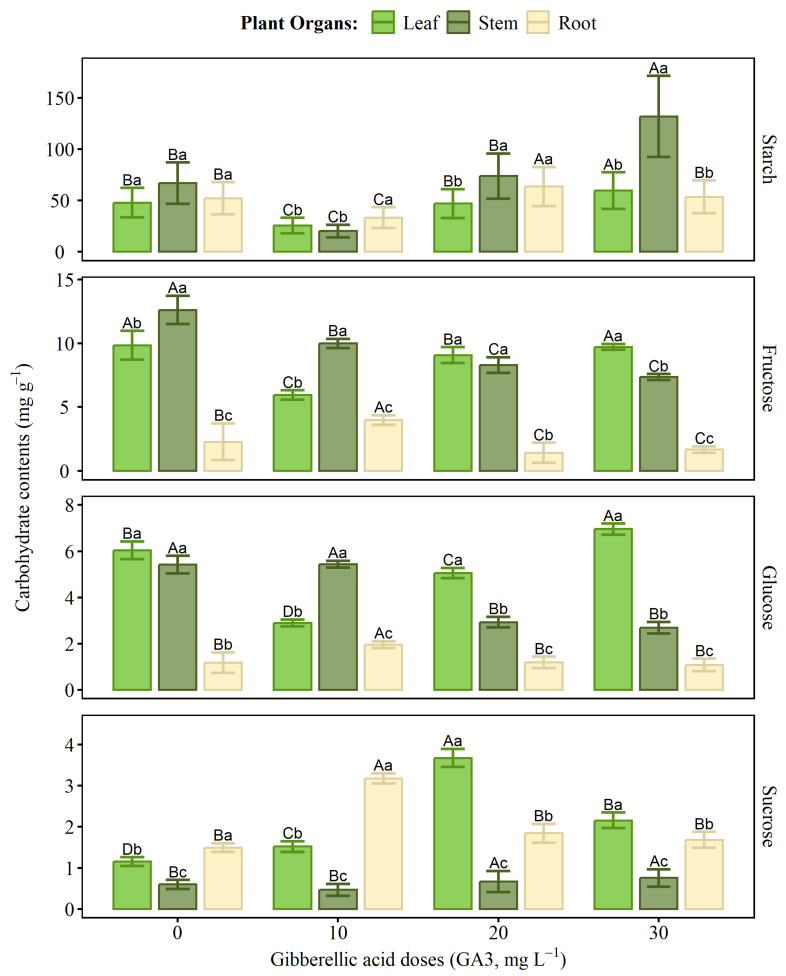
Starch, fructose, glucose, and sucrose contents in leaves, stems, and roots of plants treated with increasing doses of gibberellic acid (GA_3_). Bars indicate the 95% confidence intervals. Capital letters compare tissues between doses, while lowercase letters compare tissues within each dose. Identical letters indicate no statistical difference by the Scott–Knott test (*p* ≤ 0.05).

The application of gibberellic acid (GA_3_) influenced the levels of soluble sugars (fructose, glucose, and sucrose) in different vegetative organs (Figure 10). Starch content varied significantly among the different plant organs in response to GA_3_ doses. The stem showed the highest starch accumulation at the 30 mg L^−1^ dose, with a statistical difference compared to the other treatments and organs. In the leaves, starch levels remained relatively stable between doses, with a slight increase at the 10 and 20 mg L^−1^ concentrations but a reduction at the highest dose. In the roots, starch content increased significantly at the 10 mg L^−1^ dose, presenting the highest values for this organ, followed by a decrease at the 20 and 30 mg L^−1^ doses.

Glucose and fructose levels showed similar patterns within each organ. In leaves, both sugars decreased at doses of 10 and 20 mg L^−1^, with a subsequent increase at 30 mg L^−1^, which resulted in the highest values. In stems, levels decreased progressively with increasing GA_3_ doses, being highest in the control (0 mg L^−1^) and lowest at 30 mg L^−1^. In roots, both glucose and fructose increased at 10 mg L^−1^ but decreased at subsequent doses.

Sucrose showed distinct patterns among organs. In leaves and roots, levels increased at doses of 10 and 20 mg L^−1^, followed by a decrease at the highest dose (30 mg L^−1^). In stems, however, sucrose accumulation was greatest at doses of 20 and 30 mg L^−1^, with levels higher than those observed in the control, although lower than those of the other carbohydrates.

**Figure 11:**
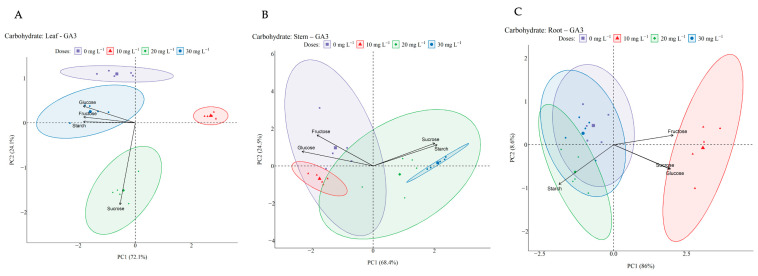
Distribution of carbohydrate levels in black pepper leaves (**A**), stems (**B**), and roots (**C**) under different gibberellin (GA_3_) doses, according to principal component analysis (PCA). The symbols represent the applied doses: 0 mg L^−1^ (purple squares), 10 mg L^−1^ (red triangles), 20 mg L^−1^ (green diamonds), and 30 mg L^−1^ (blue circles). The ellipses indicate the dispersion of the treatments, while the arrows represent the correlation of carbohydrates (sucrose, glucose, fructose, and starch) with the principal components (PC1 and PC2).

In black pepper leaves, non-structural carbohydrate contents were influenced by different GA_3_ doses, as demonstrated by principal component analysis (Figure 11A). PCA showed that the 10 mg L^−1^ dose presented a distinct metabolic profile, located alone in the positive quadrant of PC1, with less association with the evaluated carbohydrates, while the 20 mg L^−1^ dose was associated with greater sucrose accumulation. The 30 mg L^−1^ dose was related to higher concentrations of glucose, fructose, and starch. The control treatment (0 mg L^−1^) formed a separate cluster, indicating a differentiated pattern in carbohydrate allocation. The separation between treatments along the principal axes indicated significant variations in the distribution and metabolism of leaf carbohydrates in response to GA_3_.

In the stem, carbohydrate content analysis also revealed marked differences between GA_3_ treatments (Figure 11B). PCA revealed a clear separation between groups, with sucrose and starch more associated with plants treated with 30 mg L^−1^, while glucose and fructose were more associated with plants without GA_3_ application (0 mg L^−1^). Furthermore, plants subjected to the 10 mg L^−1^ dose formed a distinct cluster, suggesting an intermediate carbohydrate profile in this organ.

PCA of carbohydrate contents in black pepper roots under different GA_3_ doses (Figure 11C) revealed a distinction between treatments along the first principal component (PC1), which explained 86% of the data variability, while the second principal component (PC2) accounted for 8.6%. The 10 mg L^−1^ dose stood out from the other treatments in the graph, showing greater proximity to soluble carbohydrates (glucose, fructose, and sucrose), while the 0 mg L^−1^ and 20 mg L^−1^ doses clustered in a region associated with starch. The 30 mg L^−1^ dose occupied an intermediate position between the groups. These results indicate variations in the carbohydrate profile of roots in response to GA_3_ application.

## 3. Discussion

### 3.1. Effects on Phenology and Floral Induction

The highest efficiency in inflorescence production was observed at a dose of 20 mg L^−1^ in stage E1. This was because the application of GA_3_ in the early stages of plant development promotes greater floral differentiation, while in more advanced stages the effect may be reduced or even inhibited [23,24]. In the early stages of plant development, buds still maintain high physiological plasticity and are more sensitive to hormonal signaling, favoring the activation of genes related to energy [25,26]. However, in stage E4, the doses of GA_3_ did not result in a significant increase in the number of inflorescences, which may be related to the limited action of GA_3_ due to the predominance of other growth regulators, such as abscisic acid and cytokinins, which are involved in the regulation of fruit growth and maturation [3].

Studies show that the exogenous use of gibberellins, such as GA3, in perennial species, such as black pepper, can positively interfere with the synthesis of the *TERMINAL FLOWER 1* (*TFL1*) gene, which has a flowering suppression function [13,27,28,29]. Flowering inhibition has been observed in perennial species such as apple, roses, saffron, and grapevines treated with gibberellin [30,31,32,33,34]. These studies demonstrate that the use of exogenously applied gibberellin can influence the expression of the TFL1 gene, altering the flowering time of perennial species and modulating plant architecture [35,36]. This finding is important because it allows for the standardization of flowering and consequently the production of black pepper to more suitable periods, ensuring greater profitability and sustainability of crops.

### 3.2. Effects on Photosynthetic Parameters

The elevation of chlorophyll a fluorescence parameters in the first cycle, especially at the 10 mg L^−1^ dose, suggests an increase in the efficiency of light energy capture and transport, possibly associated with the regulation of photosystem II by GA_3_ [37]. In the second cycle, most parameters showed a reduction, which may indicate a physiological adaptation mechanism or a residual effect of the growth regulator [38]. Furthermore, energy dissipation did not undergo significant changes, which may be related to the maintenance of photochemical protection mechanisms even in the face of variations in light absorption [39]. This response may indicate an adjustment in electron flow in the transport chain as an adaptive mechanism in the face of possible environmental stress. This behavior suggests the occurrence of partial photoinhibition or a physiological adaptation aimed at optimizing energy production under less favorable conditions [40].

The temporal response of chlorophyll a fluorescence also followed a dynamic pattern, with increased absorption and energy capture up to 56 days after GA_3_ application, followed by a reduction at 70 days. Doses of 10 and 20 mg L^−1^ demonstrated greater efficiency in the use of light energy, while 30 mg L^−1^ did not provide additional benefits, indicating a possible saturation in the response to GA_3_, as already observed in studies on the regulation of photosynthesis by plant hormones [41].

For photosynthetic pigments, the application of GA_3_ influenced the levels of chlorophyll a, chlorophyll b, and total chlorophyll, with variations over time and between the evaluated cycles, with higher values recorded in the first cycle compared to the second cycle, regardless of the doses. Furthermore, the dose of 30 mg L^−1^ in the second cycle resulted in the greatest reduction in chlorophyll, suggesting a possible inhibitory effect at high concentrations, potentially associated with the induction of leaf senescence. Chlorophyll b showed greater variation over time, which may indicate greater sensitivity of this pigment to the action of GA_3_, directly reflecting the reduction in total chlorophyll.

### 3.3. Effects on Growth Characteristics and Biomass Allocation

Principal component analysis (PCA) highlighted a greater influence of shoot variables on the total variability of the data, indicating that GA_3_ acts primarily on aboveground growth, with indirect effects on the root system. The increase in the number of leaves, leaf area, and leaf dry mass indicates greater photosynthetic efficiency and seedling vigor. In the stem, GA_3_ favored growth and robustness, as well as the length of the cutting. In the root system, there was an increase in root dry mass but without an effect on the number and length of roots. The differentiated response of the analyzed variables suggests that GA_3_ acts primarily in the allocation of biomass to the shoots, promoting more significant growth in the leaves and stem compared to the root system [42]. This effect may be beneficial for accelerating the initial development of seedlings. However, disproportionate growth of roots and shoots can affect the capacity for water and nutrient absorption, compromising the plant’s adaptation in the field.

### 3.4. Effects on Non-Structural Carbohydrates

The application of GA_3_ significantly influenced the distribution of non-structural carbohydrates in different organs of the black pepper plant, highlighting its regulatory role in the plant’s energy metabolism. Previous studies indicated that GA_3_ can modulate carbon allocation by stimulating the translocation and accumulation of carbohydrates in different plant tissues [43]. In black pepper leaves, fructose showed the highest levels, while glucose and sucrose levels varied according to the GA3 dose, with a notable accumulation of sucrose at 20 mg L^−1^ and glucose and starch at 30 mg L^−1^. This occurs because GA_3_ can promote phloem efficiency and the expression of sugar transporters, which may favor the energy supply for reproductive processes such as flowering and initial fruit development [44].

In the roots, fructose was the predominant carbohydrate at all doses, with the highest levels recorded at 10 and 20 mg L^−1^, while glucose showed a slight reduction with increasing GA_3_ concentration. This pattern may be related to the fact that GA_3_ promotes the allocation of carbohydrates to underground organs [45]. Furthermore, the 10 mg L^−1^ dose was more associated with the presence of soluble sugars, while higher doses favored starch accumulation, suggesting a possible redirection of carbohydrates for energy storage, a phenomenon also observed in other crops under the action of growth regulators [46]. In the stem, a progressive reduction in fructose and glucose levels was observed as the dose increased, while sucrose remained unchanged, with the 30 mg L^−1^ dose favoring starch and sucrose accumulation in the stem.

Thus, although the data found regarding the effect of GA_3_ on growth, development, photosynthesis, and non-structural carbohydrates in black pepper plants of the Bragantina cultivar provide valuable information, some limitations must be acknowledged. The evaluations were conducted in a single location and under specific environmental conditions and a controlled environment, using a single cultivar, and no molecular analyses were performed, which may limit the generalizability of the results. Therefore, future tests should include trials in multiple locations, in a field environment, with long-term studies, including plants at different growing ages and with comparisons between different cultivars.

## 4. Materials and Methods

### 4.1. Experimental Area, Design, and Growing Conditions

The experiment was conducted at the Capixaba Institute for Research, Technical Assistance, and Rural Extension (INCAPER) in Linhares, Espírito Santo (south latitude: 19°23′28″, west longitude: 40°04′20″; altitude: 33 m). The climate is classified as Aw, tropical rainy, with a dry season in winter. The average temperature of the coldest month is above 18 °C, and the average precipitation of the driest month is less than 60 mm [47].

**Figure A2:**
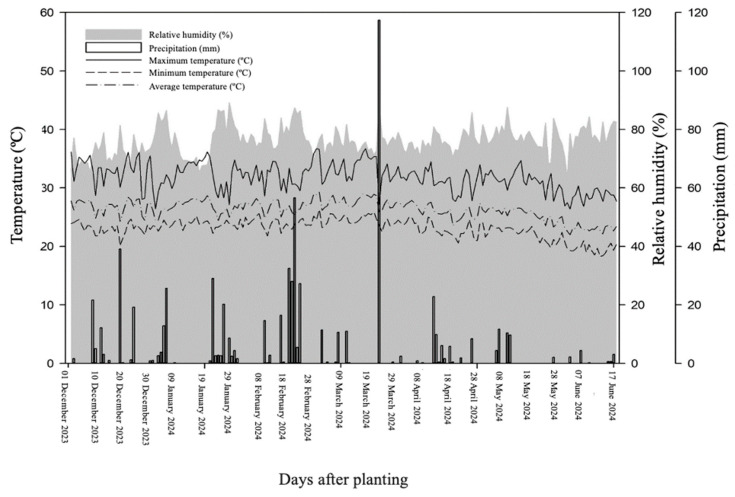
Total precipitation (mm); relative humidity (%); and maximum, average, and minimum air temperatures recorded at the Linhares-ES meteorological station from December 2023 to June 2024.

Monthly data for temperature (minimum, maximum, and average) in °C, precipitation (mm), and relative humidity (%) for the period from December 2023 to June 2024 were obtained from the Linhares-ES Automatic Meteorological Station. These data were obtained from the National Institute of Meteorology (INMET) and are presented in Figure A2.

Black pepper seedlings of the Bragantina cultivar, internationally known as ‘Panniyur-1’ [48], were used. The seedlings were obtained from local commercial nurseries and propagated by cuttings. Five months after staking, the seedlings were transplanted into 7 L plastic pots containing a commercial substrate (tropstrate HT) and thirty grams of osmocote, with the seeds released every 5–6 months per pot, as recommended by Alexandre et al. [49]. When symptoms of nutritional deficiency were detected, diagnosed through leaf analysis, microscopy, and visual analysis, foliar fertilization was performed with Captan SC nutrient solution (480 g/L of captan), formulated by Adama S.A. (Londrina, Paraná, Brazil), as recommended by nurserymen producing this crop seedling. The plants were grown in full sun under micro-sprinkler irrigation and maintained in this environment throughout the experiment.

Floral induction occurred in two cycles: the first cycle began three months after transplanting the seedlings (8-month-old plants), and the second cycle began six months after transplanting (11-month-old plants) (Table A5). Four concentrations of Gibberellic acid GA_3_ (0, 10, 20, and 30 mg L^−1^) were analyzed, applied via foliar application. The experiment was conducted in a randomized complete block design with six replicates of 56 seedlings, totaling 336 plants. Applications were made at the end of the day using a 20 L handheld backpack sprayer.

### 4.2. Flowering and Phenological Classification

**Figure A3:**
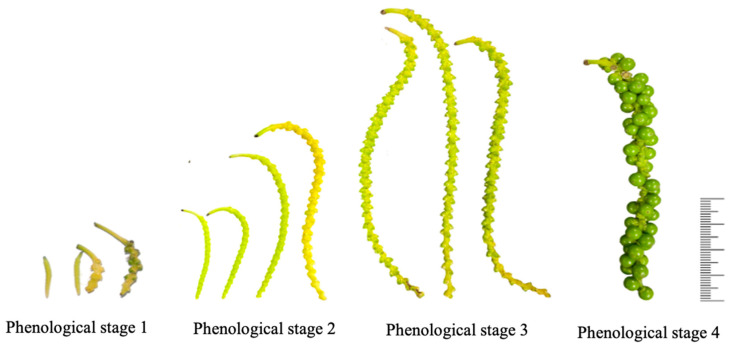
Phenological classification scale for black pepper inflorescences: phenological stage 1 is immature spikes (E1); phenological stage 2 is spikes with flowers (E2); phenological stage 3 is spikes with immature berries (E3); and phenological stage 4 is fully mature spikes with green berries (E4).

Flowering monitoring was carried out using the same procedures as those used by Silva et al. [50], which consisted of visual observations of inflorescence emergence using a magnifying glass. Assessments were made weekly two weeks after GA_3_ application. Phenological classification occurred at the end of each cycle, when the inflorescences were removed and classified according to their respective phenological stages, as per Lekha et al. [51], where E1 denotes immature spikes, E2 denotes spikes with flowers, E3 denotes spikes with immature berries, and E4 denotes fully mature spikes with green berries (Figure A3).

### 4.3. Chlorophyll a Fluorescence

During the experiment, chlorophyll a fluorescence assessments were performed from 8:00 to 10:30 a.m. using a Pocket-PEA fluorometer (Hansatech, Norfolk, UK), following the guidelines of Strasser et al. [52]. Two fully expanded leaves were dark-acclimated using leaf clips for 30 min to ensure complete photosystem oxidation. Then, a saturating light pulse of 3000 μmol m^−2^ s^−1^ of photons, lasting 1 s, was applied, and the parameters were subjected to the JIP Test (Table A6).

### 4.4. Chlorophyll Index

Chlorophyll index measurements were performed biweekly after GA_3_ application, using an electronic chlorophyll meter (ClorofiLOG, model CFL 1030, Porto Alegre, Brazil) to determine chlorophyll a and b and total chlorophyll indices [53]. These measurements were taken on a fully expanded leaf from the middle third of the plant, located on the portion facing the morning sun. Measurements were taken between 8:00 and 10:00 a.m., using the same leaf previously marked for chlorophyll fluorescence measurements.

### 4.5. Growth and Biomass Allocation Analysis

Seven months after the first product application, measurements were taken of stem diameter using a digital caliper; stem and root length, leaf number, and leaf area using a LI-COR 3100 m (LI-COR Environmental, Lincoln, NE, USA); and root volume using water displacement in a test tube. The dry mass of vegetative and reproductive organs was determined using a precision analytical balance after drying in an oven with forced-air circulation at 65 °C until a constant dry mass was obtained.

From these data, we calculated the specific leaf area (SLA: fresh leaf area divided by leaf dry mass—SLA = LA/LDM). Stem mass fraction (SLM: stem dry mass divided by total plant dry mass—SLM = STDM/TDM), expressed in g g^−1^, was measured according to Poorter et al. [54]. Root mass fraction (RMF: root dry mass divided by total plant dry mass—RMF = RDM/TDM) was calculated according to Poorter et al. [54], with the results expressed in g g^−1^. Specific root length (SRL: root length divided by root dry mass—SRL = RL/RDM), expressed in m g^−1^, was measured according to Kramer-Walter et al. [55]. Root tissue density (RTD: root dry mass divided by fresh root volume—RTD = RDM/FRV) was measured as described by Kramer-Walter et al. [55], with values expressed in g cm^−3^. Robustness index (RI: stem length divided by stem diameter—RI = SL/SD) and Dickson quality index (DQI: ratio between total dry mass and the sum of two proportions—DQI = TDM/[(SL/SD) + (SDM/RDM)]) were calculated. The specific shoot length (SSL) was calculated from the ratio between the stem length and the stem dry mass (SSL = SL/STDM), with the results expressed in m g^−1^, as described by Poorter et al. [54].

### 4.6. Non-Structural Carbohydrates

For the analysis of soluble carbohydrate and starch contents, at the end of each cycle, samples of leaves, stems, and roots were inactivated in a microwave at 600 watts for approximately 90 s [56]. The samples were then dried in a forced-air oven at 65 °C until a constant mass was obtained, followed by pulverization in a ball mill (model: TE-350; TECNAL, São Paulo, Brazil) for 3 min. This process was repeated for an additional 3 min, totaling approximately 6 min, depending on the hardness of the material.

The extraction of soluble carbohydrates followed the method described by Pollock [57], performed through four extractions using 80% ethanol. In the first step, 1.5 mL of 80% ethanol was added to the tube containing the previously weighed samples. The mixture was then homogenized using a vortex. For the aforementioned analysis, a High-Performance Anion Exchange Chromatography (HPLC) system was used on a Shimadzu SIL-10AF chromatograph (Shimadzu Corporation, Kyoto, Japan). Separation was performed with a Shim-Pack^®^ SPR-Pb column (Shimadzu Corporation, Kyoto, Japan) (250 × 7.8 mm), using ultrapure water as the mobile phase, at a flow rate of 0.6 mL/min, a column temperature maintained at 80 °C, and detection by refractive index. Sugar identification and quantification were based on commercial standards from Sigma-Aldrich^®^ (Merck, Jacarepaguá, Rio de Janeiro, Brazil).

Starch quantification was performed using an enzymatic method, as described by Amaral et al. [58]. For enzyme preparation, α-amylase was diluted in MOPS buffer at a concentration of 120 U/mL, while amyloglucosidase was diluted in sodium acetate buffer at a concentration of 30 U/mL. To the dried precipitate of the samples, 0.5 mL of the α-amylase solution was initially added, followed by incubation in a water bath at 75 °C for 30 min. Then, another 0.2 mL of the same enzyme was added, with a further incubation for 30 min at 75 °C. After the enzymatic step, the samples were read on an ELISA plate to quantify the sugars.

### 4.7. Statistical Analysis

The data were subjected to the Shapiro–Wilk normality test to assess the distribution of variables. Data transformations were performed according to Box et al. [59], using the cubic root (cbrt), inverse, logarithmic (log), and square root (sqrt) functions, as needed, to adjust the distribution.

Analysis of variance was performed for normally distributed data, and the Scott–Knott test (*p* < 0.05) was applied to variables that showed a significant difference using the F test. The Student’s t-test (*p* ≤ 0.05) was used for chlorophyll a fluorescence parameters. Principal component analysis (PCA) was used for variables related to growth and non-structural carbohydrates. Statistical analyses were performed using R statistical software, version 4.0.2, and R Studio 3.0.1.

For non-structural carbohydrates, a randomized block design was adopted, organized in a triple factorial scheme (4 × 3 × 4). The first factor corresponded to the doses (0, 10, 20, and 30 mg L^−1^), the second factor to the plant organs (leaf, stem and root) and the third factor to the types of carbohydrates analyzed (starch, fructose, glucose and sucrose).

## 5. Conclusions

The application of gibberellic acid (GA_3_) significantly influenced the flowering, physiology, growth, and metabolism of black pepper. Intermediate doses (10 and 20 mg L^−1^) favored floral induction in early stages, increased photochemical efficiency, and stimulated the production of soluble carbohydrates, while the 30 mg L^−1^ dose promoted greater vegetative growth, with biomass accumulation in shoots and roots. Chlorophyll levels and chlorophyll a fluorescence parameters indicated improved photosynthetic activity in the early stages after application, especially in the first cycle. Furthermore, GA_3_ modulated the distribution of sugars between tissues, particularly the accumulation of sucrose in leaves and starch in stems and roots. These results reinforce that the appropriate definition of the dose and time of application is essential to enhance the desired effects on flowering and the physiological and productive performance of the crop.

## Data Availability

The original contributions presented in this study are included in the article. Further inquiries can be directed to the corresponding authors.

## Abbreviations

The following abbreviations are used in this manuscript:

ABS/CS_0_	Energy absorption per reaction center
DI_0_/CS_0_	Energy dissipation per reaction center
TR_0_/CS_0_	Energy capture by the reaction center
ET_0_/CS_0_	Electron transport rate beyond QA^−^
RE_0_/CS_0_	Reduction of final acceptors in the electron transport chain
φP_0_	Quantum efficiency of primary photochemistry
φE_0_	Quantum efficiency of electron transport
NL	Number of leaves
LDM	Leaf dry mass
LA	Leaf area
SL	Stem length
STL	Shoot length
IQD	Dickson quality index
RDM	Root dry mass
TDM	Total dry mass
SDM	Shoot dry mass
STDM	Stem dry mass
RMF	Root mass fraction
LMF	Leaf mass fraction
SRL	Specific root length
SD	Stem diameter
SLA	Specific leaf area
SLM	Stem mass fraction
RL	Root length
RTD	Root tissue density
FRV	Fresh root volume
RI	Robustness index
SSL	Specific shoot length

## Appendix A

**Table A1:** Summary of the analysis of variance for flowering variables in black pepper plants subjected to different treatments with GA_3_. The sources of variation (SVs), degrees of freedom (DFs), and mean squares for the number of inflorescences in black pepper plants, cultivar Bragantina, are presented.

SV	Mean Square	
	DF	Number of Inflorescences
Block	5	8.933 ns
Doses GA_3_	3	177.556 ***
Stage	3	151.333 ***
Cycle	1	0.75 ns
Doses GA_3_:Stage	9	224.481 ***
Doses GA_3_:Cycle	3	1.639 ns
Stage:Cycle	3	4.75 ns
Doses GA_3_:Stage:Cycle	9	8.046 ns
Residue	155	20.662
CV	(%)	237.16

Coefficient of variation (CV). Significance levels: *** *p* < 0.001; ns = not significant.

**Table A2:** Summary of the analysis of variance for variables related to chlorophyll fluorescence in black pepper leaves subjected to doses of GA_3_, days after application (DAA) and cycle. The sources of variation (SVs), degrees of freedom (DFs), and mean squares of the characteristics ABS/CS_0_ (energy absorption by reaction center), DI_0_/CS_0_ (energy dissipation), ET_0_/CS_0_ (electron transport rate), RE_0_/CS_0_ (final reduction in the electron transport chain), TR_0_/CS_0_ (energy capture by the reaction center), ΦE_0_ (electron transport efficiency to plastoquinone), ΦP_0_ (quantum efficiency of photosystem II), and ΦR_0_ (reduction efficiency of final acceptors in the electron transport chain) in black pepper plants, cultivar Bragantina, are presented.

SV	Mean Squares								
	DF	ABS/CS_0_	DI_0_/CS_0_	ET_0_/CS_0_	RE_0_/CS_0_	TR_0_/CS_0_	ΦE_0_	ΦP_0_	ΦR_0_
Block	5	26,485,825.851 ***	4,106,172.813 **	894,265.998 *	330,550.868 ***	4,109,675.094 ***	0.084 ***	0.018 *	0.033 ***
Doses GA_3_	3	5,267,797.562 ns	40,933.384 ns	4,184,462.425 ***	172,900.702 ***	1,057,381.073 ns	0.032 ns	0.017 ns	0.004 ns
DAA	5	683,155,636.878 ***	324,914,236.462 ***	50,648,245.801 ***	4,745,723.598 ***	81,423,035.701 ***	2.091 ***	0.728 ***	1.419 ***
Cycle	1	45,675,055.072 ***	3,151,449.204 ns	2,052,733.07 *	2,669,360.852 ***	10,153,270.518 ***	0.007 ns	0.024 ns	0.328 ***
Doses GA_3_:DAA	15	8,411,235.37 ***	5,309,972.64 ***	1,776,368.051 ***	120,478.651 ***	2,135,646.223 ***	0.019 ns	0.009 ns	0.01 **
Doses GA_3_:Cycle	3	3,813,783.896 ns	2,607,688.847 ns	748,670.067 ns	5204.722 ns	735,942.319 ns	0.023 ns	0.025 *	0.005 ns
DAA:Cycle	4	27,093,867.343 ***	925,721.717 ns	9,182,002.748 ***	4,172,154.025 ***	10,989,157.144 ***	0.664 ***	0.009 ns	0.793 ***
Doses GA_3_:DAA:Cycle	12	2,629,592.182 ns	1,278,313.846 ns	395,742.008 ns	16,885.352 ns	570,538.165 ns	0.013 ns	0.024 ***	0.003 ns
Residue	976	3,088,608.392	1,036,307.182	401,486.746	26,949.49	658,165.291	0.014	0.007	0.005
CV	(%)	21.45	38.16	37.08	26.09	14.91	40.68	12.66	52.01

Coefficient of variation (CV). Significance levels: *** *p* < 0.001, ** *p* < 0.01, * *p* < 0.05; ns = not significant.

**Table A3:** Summary of the analysis of variance with the sources of variation (SVs), degrees of freedom (DFs) and mean squares (MSs) for chlorophyll A, chlorophyll B and total chlorophyll subjected to doses of GA_3_, days after application (DAA) and cycle, in black pepper plants, cultivar Bragantina.

SV	Mean Squares			
	DF	Chlorophyll A	Chlorophyll B	Total Chlorophyll
Block	5	1168.435 ***	2333.116 ***	3172.836 ***
Doses GA_3_	3	769.422 ***	575.165 ***	2815.192 ***
DAA	3	1468.81 ***	1887.476 ***	5245.412 ***
Cycle	1	49,562.155 ***	66,448.513 ***	213,134.052 ***
Doses GA_3_:DAA	9	447.916 ***	99.164 ns	997.325 ***
Doses GA_3_:Cycle	3	139.344 ns	48.368 ns	314.234 ns
DAA:Cycle	3	542.171 ***	1410.83 ***	4843.249 ***
Doses GA_3_:DAA:Cycle	9	827.512 ***	434.15 ***	1584.761 ***
Residue	2651	60.479	77.586	279.686
CV	(%)	20.93	57.23	31.72

Coefficient of variation (CV). Significance levels: *** *p* < 0.001; ns = not significant.

**Table A4:** Summary of the analysis of variance for variables related to the vegetative development of the root system of black pepper, cultivar Bragantina, subjected to doses of gibberellin (GA_3_) (0, 10, 20 and 30 mg/L) with the sources of variation (SVs), degrees of freedom (DFs) and mean squares for the characteristics leaf area (LA) (cm^2^), leaf mass fraction (LMF) (%), Dickson quality index (DQI), leaf dry mass (LDM) (g), shoot dry mass (SDM) (g), number of leaves (NL), shoot length (STL) (cm), stem length (SL) (cm), stem mass fraction (SLM) (%), stem dry mass (STDM) (g), total dry mass (TDM) (g), specific root length (SRL), root mass fraction (RMF) (%), SDM/RDM ratio, and root dry mass (RDM) (g) in black pepper plants, cultivar Bragantina.

SV		Mean Square							
	DF	LA	LMF	DQI	LDM	SDM	NL	STL	SL
Block	5	327.50 ***	39,892 ***	1633 *	298.00 ***	20.41 **	458.10 **	544.50 ns	115.50 ns
Doses GA_3_	3	162.70 **	1310 **	3336 **	401.30 **	29.79 ***	721.90 **	1237.00 *	138.90 **
Residue	327	38.24	0.6779	6177	42.91	5125	131.00	323.50	43.58
CV	(%)	21.02	21.5	40.54	24.16	40.54	36.47	28.47	26.65
		SLM	STDM	TDM	SRL	RMF	RDM	SDM/RDM	
Block	5	2430 ***	5131 *	243.50 **	0.041 **	0.009 *	0.071 ns	2054 ns	
Doses GA_3_	3	2102 *	9232 **	370.70 **	0.042 *	0.009 *	0.272 ***	3336 *	
Residue	327	5599	1747	77.38	0.013	0.003	0.048	1.081	
CV	(%)	16.05	44.21	19.79	64.71	36.79	15.6	51.53	

Coefficient of variation (CV). Significance levels: *** *p* < 0.001, ** *p* < 0.01, * *p* < 0.05; ns = not significant.

**Table A5:** Evaluation period, cycle duration and number of gibberellin (GA_3_) applications on black pepper, cv. Bragantina.

Cycle	Months of Evaluation	Cycle Duration		Number of Applications
		Start	End	
1	December to February	1 December 2023	17 February 2024	1
2	February to May	18 February 2024	18 May 2024	1

**Table A6:** Parameter abbreviations, formulas, and descriptions of data derived from chlorophyll a fluorescence transients.

Parameter	Formulas	Description
ABS/CS_0_	Chl/CS	Energy absorption per reaction center
DI_0_/CS_0_	ABS/RC − TR_0_/RC	Energy dissipation per reaction center
TR_0_/CS_0_	ϕP_0_ (ABS/CS)	Energy capture by the reaction center
ET_0_/CS_0_	ϕP^0^*Ψ_0_*(ABS/CS)	Electron transport rate beyond QA^−^
RE_0_/CS_0_	(RE_0_/ET_0_) − ET_0_/CS_0_)	Reduction of final acceptors in the electron transport chain
φP_0_	TR_0_/ABS = [1 − (F_0_/Fm)] = FV/Fm	Quantum efficiency of primary photochemistry
φE0	ET_0_/ABS = [1 − (F_0_/Fm)] ψ_0_	Quantum efficiency of electron transport
